# Associations between Household-Level Exposures and All-Cause Diarrhea and Pathogen-Specific Enteric Infections in Children Enrolled in Five Sentinel Surveillance Studies

**DOI:** 10.3390/ijerph17218078

**Published:** 2020-11-02

**Authors:** Josh M. Colston, Abu S. G. Faruque, M. Jahangir Hossain, Debasish Saha, Suman Kanungo, Inácio Mandomando, M. Imran Nisar, Anita K. M. Zaidi, Richard Omore, Robert F. Breiman, Samba O. Sow, Anna Roose, Myron M. Levine, Karen L. Kotloff, Tahmeed Ahmed, Pascal Bessong, Zulfiqar Bhutta, Estomih Mduma, Pablo Penatero Yori, Prakash Sunder Shrestha, Maribel P. Olortegui, Gagandeep Kang, Aldo A. M. Lima, Jean Humphrey, Andrew Prendergast, Francesca Schiaffino, Benjamin F. Zaitchik, Margaret N. Kosek

**Affiliations:** 1 Division of Infectious Diseases and International Health, University of Virginia School of Medicine, Charlottesville, VA 22903, USA; josh.colston@virginia.edu (J.M.C.); pyori@virginia.edu (P.P.Y.); 2 Centre for Nutrition & Food Security, International Centre for Diarrhoeal Disease Research, Dhaka 1212, Bangladesh; gfaruque@icddrb.org; 3 Medical Research Council Unit—The Gambia at the London School of Hygiene & Tropical Medicine, Banjul P.O. Box 273, Republic of Gambia; jhossain@mrc.gm; 4 Epidemiology and Health Economics, GSK Vaccines, 1300 Wavre, Belgium; debasish.x.saha@gsk.com; 5 Suman Kanungo—National Institute of Cholera and Enteric Diseases, Kolkota 700010, India; sumankanungo@gmail.com; 6 Centro de Investigação em Saúde de Manhiça, Manhica CP 1929, Mozambique; inacio.mandomando@manhica.net; 7 Department of Pediatrics and Child Health, The Aga Khan University, Karachi 74800, Pakistan; imran.nisar@aku.edu (M.I.N.); anita.zaidi@aku.edu (A.K.M.Z.); 8 Kenya Medical Research Institute, Center for Global Health Research, Kisumu, Nyanza 40100, Kenya; omorerichard@gmail.com; 9 Hubert Department of Global Health, Rollins School of Public Health, Emory University, Atlanta, GA 30322, USA; rfbreiman@emory.edu; 10 Centre pour le Développement des Vaccins, Bamako BP 251, Mali; ssow@som.umaryland.edu; 11 Indiana University School of Medicine, Indianapolis, IN 46202, USA; awroose@iu.edu; 12 Departments of Medicine and Pediatrics, Center for Vaccine Development and Global Health, University of Maryland School of Medicine, Baltimore, MD 21201, USA; mlevine@som.umaryland.edu; 13 Department of Pediatrics, Center for Vaccine Development and Global Health, University of Maryland School of Medicine, Baltimore, MD 21201, USA; kkotloff@som.umaryland.edu; 14 Nutrition and Clinical Services Division, International Centre for Diarrhoeal Disease Research, Bangladesh (icddr,b), Dhaka 1212, Bangladesh; tahmeed@icddrb.org; 15 HIV/AIDS & Global Health Research Programme, University of Venda, Thohoyandou, Limpopo 0950, South Africa; Pascal.Bessong@univen.ac.za; 16 Department of Pediatrics and Child Health, Aga Khan University, Karachi 74800, Pakistan; zulfiqar.bhutta@aku.edu; 17 Haydom Global Health Institute, Haydom P.O. Box 9000, Tanzania; estomduma@gmail.com; 18 Department of Child Health, Institute of Medicine of Tribhuvan University, Kirtipur 44618, Nepal; prakashsunder@hotmail.com; 19 Asociacion Benefica PRISMA, Iquitos 16006, Peru; mparedeso@prisma.org.pe; 20 Department of Gastrointestinal Sciences, Christian Medical College, Vellore 632004, India; gkang@cmcvellore.ac.in; 21 Department of Physiology and Pharmacology, Faculty of Medicine, Federal University of Ceará, Fortaleza 60020-181, Brazil; alima@ufc.br; 22 Department of International Health, Johns Hopkins Bloomberg School of Public Health, Baltimore, MA 21205, USA; jhumphr2@jhu.edu; 23 Centre for Paediatrics, Blizard Institute, Queen Mary University of London, London E1 2AT, UK; a.prendergast@qmul.ac.uk; 24 Faculty of Veterinary Medicine, Universidad Peruana Cayetano Heredia, Lima 15102, Peru; francesca.schiaffino@upch.pe; 25 Department of Earth and Planetary Sciences, Johns Hopkins Krieger School of Arts and Sciences, Baltimore, MA 21218, USA; zaitchik@jhu.edu; 26 Division of Infectious Diseases, International Health and Public Health Sciences, Department of Internal Medicine, University of Virginia, Charlottesville, VA 22903, USA

**Keywords:** enteropathogens, water, sanitation and hygiene, child health, diarrheal disease, zoonoses

## Abstract

Diarrheal disease remains a major cause of childhood mortality and morbidity causing poor health and economic outcomes. In low-resource settings, young children are exposed to numerous risk factors for enteric pathogen transmission within their dwellings, though the relative importance of different transmission pathways varies by pathogen species. The objective of this analysis was to model associations between five household-level risk factors—water, sanitation, flooring, caregiver education, and crowding—and infection status for endemic enteric pathogens in children in five surveillance studies. Data were combined from 22 sites in which a total of 58,000 stool samples were tested for 16 specific enteropathogens using qPCR. Risk ratios for pathogen- and taxon-specific infection status were modeled using generalized linear models along with hazard ratios for all-cause diarrhea in proportional hazard models, with the five household-level variables as primary exposures adjusting for covariates. Improved drinking water sources conferred a 17% reduction in diarrhea risk; however, the direction of its association with particular pathogens was inconsistent. Improved sanitation was associated with a 9% reduction in diarrhea risk with protective effects across pathogen species and taxa of around 10–20% risk reduction. A 9% reduction in diarrhea risk was observed in subjects with covered floors, which were also associated with decreases in risk for zoonotic enteropathogens. Caregiver education and household crowding showed more modest, inconclusive results. Combining data from diverse sites, this analysis quantified associations between five household-level exposures on risk of specific enteric infections, effects which differed by pathogen species but were broadly consistent with hypothesized transmission mechanisms. Such estimates may be used within expanded water, sanitation, and hygiene (WASH) programs to target interventions to the particular pathogen profiles of individual communities and prioritize resources.

## 1. Introduction

Despite considerable progress in recent years, diarrheal disease remains a major cause of mortality and morbidity in childhood [1,2]—responsible for some 446,000 under-five deaths in 2016 [3]—and its sequelae lead to poor health and economic outcomes in adulthood [4,5]. New diagnostic and statistical methods, as well as a number of ambitious multi-site, population-based studies have shed light on the pathogen-specific etiologies of diarrheal disease [6,7,8]. Numerous microbial agents each with distinct transmission dynamics interact with host-level, environmental, and meteorological risk factors to cause enteric infections which may be symptomatic or sub-clinical [9,10]. Understanding these complexities may in turn assist in the identification of tailored interventions to reduce the impact of enteric disease.

In low-resource settings, infants and young children are exposed to numerous risk factors for enteric pathogen transmission within their homes, though the relative importance of different transmission pathways vary depending on the pathogen type and species [11]. While food- and water-borne transmission are the dominant pathways for most bacteria and protozoa, enteric viruses appear to depend on more direct person-to-person contact [12], an observation supported by high rates of infection even in settings with developed water and sanitation infrastructures. Inadequate sanitation and drinking water are leading household-level risk factors for all-cause childhood diarrhea but, while systematic reviews have estimated that improvements to these facilities can reduce the risk of this outcome by between 25% and 75% [13,14], three high-profile randomized control trials found more mixed, context-dependent effects [15]. Furthermore, they likely do not impact transmission of rotavirus [16], one of the pathogens responsible for the largest share of the diarrheal disease burden [2,17,18]. Floors that are covered with wood, tiles, or cement may protect against transmission of some pathogens compared to floors made of packed earth or sand [19,20,21,22]; however, the underlying mechanisms are unclear. The link between a mother’s level of education and the health, nutrition, and mortality prospects of her offspring is credited with having averted 4.2 million child deaths between 1970 and 2009 due to increased female educational attainment [23,24,25] and associations between caregiver education and diarrheal disease outcomes have been documented [22,26]. Finally, household crowding, whereby the number of occupants of a dwelling exceeds the capacity of its space, is a risk factor for numerous adverse health conditions including diarrheal disease [27]. While several studies have attempted to characterize the associations underlying these risk factors by taxa—separately for viral, bacterial, and protozoal agents [22,28]—pathogen species-specific analyses have, until very recently, not been possible due to the prohibitive sample sizes and limitation in diagnostic capacity using culture based methods or targeted PCR for fewer than five pathogens concurrently [29].

The objective of the analysis reported here was to model the associations between five household-level risk factors—water, sanitation, floor material, caregiver education, and household crowding—and infection status for 16 common, high-burden enteric pathogens ascertained in children aged 0–5 years enrolled in five surveillance studies. The a priori hypothesis to be tested was that improved sanitation and water sources, finished floors, caregiver primary school completion, and lack of crowding are protective against enteric infection, but to an extent that differs by pathogen species and taxon.

## 2. Materials and Methods

### 2.1. Study Population

**Figure 1:**
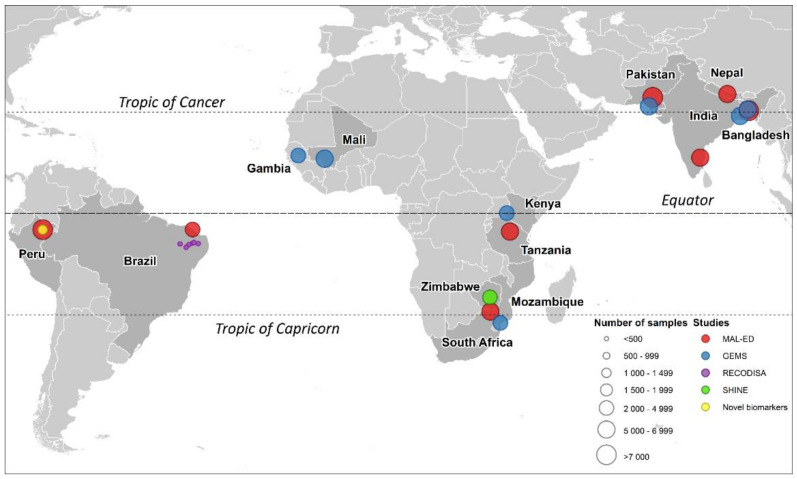
Locations of the 22 study sites in which the 5 studies were carried out and numbers of available stool samples (before exclusion of samples not diagnosed by qPCR).

This analysis combined data from five studies. Studies were considered eligible for inclusion if they used polymerase chain reaction (PCR) to diagnose the same panel of 16 enteropathogens, if they had collected data relating to all 5 exposures and if they were willing to share data for the purposes of the analysis. The Etiology Risk Factors and Interactions of Enteric Infections and Malnutrition and the Consequences for Child Health and Development (MAL-ED) project was a multi-site cohort study in which 2100 newborns (227–303 per site) were recruited from communities in eight different Low- and Middle-Income countries and were monitored continuously over their first 2 years of life [30]. At the MAL-ED site in Loreto, Peru, a shorter cohort study was carried out to evaluate novel biomarkers of environmental enteropathy (“Novel Biomarkers”). The Global Enteric Multicenter Study (GEMS) was a multi-site case-control study, in which cases of moderate-to-severe diarrhea (MSD) in children under 5 years old presenting at health facilities were matched by age, sex, and time of presentation of the index case with healthy controls recruited from the same community at sites in seven countries [31]. The Study of the Etiology of Childhood Diarrhea in the Brazilian Semiarid Region (part of the RECODISA—Rede de Ovino Caprino Cultura e Diarréia Infantil no Semi-Árido Brasileiro—network) was a case-control study carried out in six Brazilian cities in which cases with diarrhea and asymptomatic, age-matched controls were identified among children aged 2–36 months by community-based active surveillance [8]. The Sanitation Hygiene Infant Nutrition Efficacy (SHINE) Trial was a cluster-randomized, community-based trial in 2 rural districts of Zimbabwe that included an embedded sub-study of environmental enteric dysfunction, in which data and biospecimens were collected from infants longitudinally up to 18 months of age [32]. The locations of the 22 study sites are shown in Figure 1.

### 2.2. Outcome Variable

In the MAL-ED, Novel Biomarkers, and SHINE studies, stool samples were collected from the subjects by locally recruited fieldworkers according to a predefined schedule (at monthly intervals following enrollment in MAL-ED, between 1 and 30 days after enrollment in Novel Biomarkers and at 1, 3, 6, and 12 months of age in SHINE) and upon reporting of a diarrheal episode by the child’s caregiver. In the other studies, locally recruited fieldworkers collected a single stool sample from each case and their matched control subject upon enrollment (although in GEMS it was possible for the same individual to be enrolled more than once and therefore contribute multiple samples, but this occurred infrequently). Enteropathogen-specific infection status was ascertained using probe-based quantitative PCR (qPCR) assays on custom-developed TaqMan Array Cards (Thermo Fisher, Waltham, MA, USA) [33] for all stool samples in Novel Biomarkers, SHINE, and RECODISA and for large subsets of the GEMS and MAL-ED samples. Samples from MAL-ED and GEMS that were tested using other methods were excluded from this analysis for consistency given the differing diagnostic sensitivities (this meant that only pathogen data from GEMS1 and not GEMS1A were included in the analysis). For consistency across studies, where Ct values were available a cutoff value of 35 cycles was used. Infection status for each of 16 highly prevalent or endemic enteric pathogen species or pathotypes were treated as binary outcome variables as was infection status for any of the three pathogen taxa—viruses, bacteria, and protozoa. The pathogen species were: adenovirus, astrovirus, norovirus, rotavirus, sapovirus, *Aeromonas*, *Campylobacter*, enteroaggregative *Escherichia coli* (*E. coli*) (EAEC), enteropathogenic *E. coli*, ((EPEC) typical and atypical), heat-labile enterotoxigenic *E. coli* (LT-ETEC), and heat-stable ETEC (ST-ETEC), *Salmonella*, *Shigella*/enteroinvasive *E. coli* (EIEC) (qPCR uses the same gene target for these two closely related pathogens), *Cryptosporidium,* and *Giardia*. Several important pathogens (e.g., *Vibrio*, *Entamoeba*, helminths) were excluded from consideration due to there having been too few detections to fit the regression models, or due to not having been tested for in every study. To ensure that a single infection episode was not counted multiple times, *Campylobacter*- and norovirus-positive samples were excluded if they were collected within 30 days of a previous sample that was positive for the same pathogen strain without being separated by an intermediate negative sample [9]. For all other pathogens that are less associated with persistent infection, a 14-day period was used, except for the two protozoa, for which samples that were positive for the same species (*Cryptosporidium parvum* or *Cryptosporidium hominis*) or assemblage (*Giardia duodenalis* A or B) as a prior sample from the same subject were excluded unless separated by three negative samples in recognition of their association with chronic infections.

### 2.3. Covariates

The primary exposures of interest were the following binary household-level factors, selected on the basis of their hypothesized association with enteric pathogen transmission and the comparability of their definitions across the contributing studies:

- Drinking water: whether or not the subject resided in a household with access to an improved drinking water source (with potential to deliver safe water by nature of its design and construction such as piped water or protected tubewells, boreholes, dug wells, or springs) [34].
- Sanitation: whether or not the subject resided in a household with access to an improved, non-shared sanitation facility (“improved” meaning designed to hygienically separate excreta from human contact) [34].
- Flooring material: whether or not the subject resided in a household that had a covered (“improved”—rudimentary or finished) as opposed to natural (“unimproved”—earth or sand) floor [35].
- Caregiver education: a binary variable indicating whether or not the subject’s caregiver had completed primary education (≥6 completed years of schooling [25]).
- Household crowding: a binary variable indicating whether or not the subject resided in a household with 3 or more residents per bedroom [22].

Definitions of these variables used by each study were mapped as closely as possible to those used in previous publications from the World Health Organization, the Demographic and Health Surveys and others, as shown in Table S2 in the supplementary material. For subjects who had multiple assessments of these variables during follow-up, the first available value was used, for consistency with those studies that only assessed them at baseline. In addition, the following covariates were included as potential confounders:

- Site: a categorical variable indicating at which of the 22 study sites the subject was enrolled, included to adjust both for between-site differences in background pathogen transmission levels and for potential confounding engendered by differences in surveillance methods between the 5 studies.
- Sample type: whether the stool sample was collected during a diarrheal episode (cases of diarrhea in GEMS and RECODISA, diarrheal collections in MAL-ED, Novel Biomarkers and SHINE) or while the subject was asymptomatic (controls in GEMS and RECODISA, surveillance samples in MAL-ED, Novel Biomarkers and SHINE).
- Age: the subjects’ age in continuous months at the time of stool sample collection, modeled using linear, quadratic, and cubic terms to account for non-linearity of association with enteric pathogen presence.
- Feeding status: a categorical variable indicating whether the child was being exclusively breastfed, partially breastfed or had been fully weaned (no longer receiving any breastmilk) at the time of sample collection.
- Nutritional status: two binary variables indicating whether or not the child was moderately or severely stunted or underweight (respectively, a length-for-age and weight-for-age Z-score of ≤−2.0) to adjust for both the impact of nutritional status on susceptibility to infections [36] and potential unobserved confounding by socio-economic status.

Missing covariate data were imputed using predictions from either multivariate normal regression, linear mixed effects models or Cox proportional hazards models, methods that are described in detail in the Supplementary Materials.

### 2.4. Statistical Methods

For all pathogen-specific associations, modified Poisson regression models were fitted to the binary outcome in the full database using generalized linear models with cluster-robust variance estimation to calculate adjusted risk ratios (RRs) for infection for each of the five risk factors in the presence of each other and the potential confounders [37]. This analysis did not attempt to attribute diarrheal episodes to particular etiological agents, and it was therefore possible (indeed common) for the same stool sample, including diarrheal samples, to be positive for multiple pathogens or negative for all pathogens. The pooled database was treated as longitudinal in structure and the GEMS and RECODISA subjects’ statuses as cases or controls were only accounted for in the analysis insofar as they determined whether they contributed diarrheal or surveillance samples. To assess their associations with all-cause diarrhea, the household-level exposures were included with covariates in Cox proportional hazards models treating the subjects’ age as survival time, reporting of a diarrheal episode as failure events and allowing for multiple failures per subject [9]. The Cox models were fitted only to the MAL-ED data since it was the only one of the five studies that collected data in a format suitable for survival analysis. Coefficients estimated from the models were visualized in dot-and-whisker plots. The crowding variable was coded with “crowded” households as the comparison group, so that a RR estimate of <1 could be interpreted in same way as the other 4 main exposures—as a protective effect of a factor which might conceivably be the target of an intervention. As a sensitivity analysis we repeated the analysis on the data from each contributing study in turn and compared these single-study results to the pooled findings. Analyses were carried out using Stata 16 [38].

## 3. Results

Table 1 gives the number and proportion of stool samples that were positive for the different species of enteropathogens in the 22 study sites and overall. Results were available for 50,000–57,000 stool samples depending on the pathogen, and overall positivity rates varied from 1.4%—*Salmonella* spp. to 50.8%—EAEC.

Table 2 shows the prevalence of the five household-level exposures in each of the study sites (before imputation of missing values and exclusion of samples not diagnosed with qPCR and including both GEMS1 and GEMS1A subjects—percent missing data by site is shown in supplementary Table S1). There was near universal coverage of improved drinking water sources among subjects in the sites in South Asia (with the exception of Karachi, Pakistan) and Brazil (with the exception of Fortaleza), while the site at Haydom, Tanzania, had by far the lowest coverage. The proportion of subjects living in households with access to improved sanitation facilities ranged from 0.0% in Haydom, to 97.0% in Patos, Brazil. Haydom also had the lowest proportion of households with covered floors, followed by Nyanza, Kenya, and Mirzapur, Bangladesh, while in the urban sites of Brazil, India, and Mali, coverage of improved flooring was high. The highest levels of caregiver primary education completion were seen at the sites in Crato, Brazil; Midlands, Zimbabwe; and Venda, South Africa, while again prevalence for this variable was lowest in Haydom, Tanzania. The site with by far the highest prevalence of household crowding (≥3 residents per bedroom) was Basse, The Gambia, while Nyanza, Kenya and Vellore, India had the lowest levels of crowding. It was not possible to calculate the crowding variable directly for the RECODISA and SHINE study sites. In both studies respondents were asked the number of residents per household, but in SHINE, no information was collected on the number of bedrooms, and in RECODISA, the number of rooms overall was recorded, but not the number of rooms for sleeping specifically. The process for imputing these and other missing data are explained in detail in the Supplementary Materials. There was very low correlation between the five exposure variables both within sites and in the pooled data (r = −0.25 to 0.25). The prevalence of the time-varying covariates among subjects at the time of enrollment (feeding status, and moderate–severe stunting and underweight) are shown in Table S3 in the Supplementary Materials.

**Figure 2:**
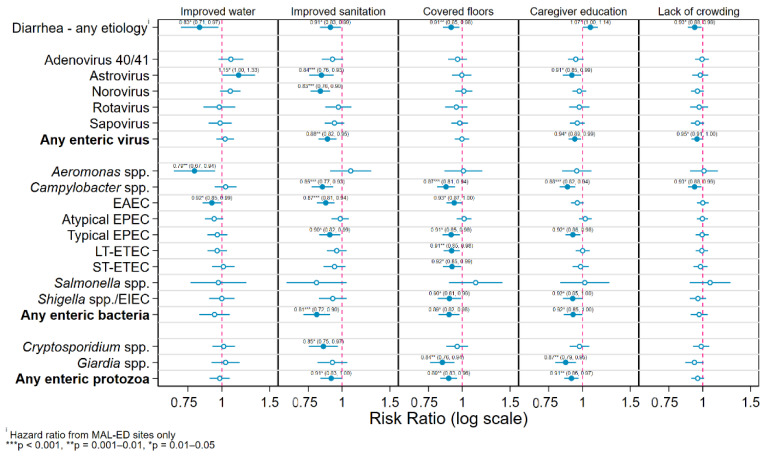
Hazard and risk ratios for detection of specific enteric pathogen species in stool samples associated with 5 household-level risk factors from Cox and generalized linear models adjusting for study site, age, sample type and feeding and nutritional status.

Figure 2 plots the RRs for detection of specific enteric pathogen species and taxa in stool samples associated with the five household-level risk factors from Cox proportional hazard and generalized linear Poisson models adjusting for study site, age, sample type and feeding and nutritional status. Living in a household with an improved drinking water source was associated with a slightly statistically significant 17% reduction in the risk of diarrhea of any etiology (hazard ratio (HR) = 0.83 (0.71, 0.97)), but a similarly significant increase in the risk of astrovirus infection of 15% (RR = 1.15 (1.00, 1.33)). Neither protozoa showed a significant association with having an improved water source; however, this variable did reduce the risk of infection with *Aeromonas* spp. by a moderately statistically significant 21% (RR = 0.79 (0.67, 0.94)) and EAEC by a slightly statistically significant 8% (RR = 0.92 (0.85, 0.99)).

Household access to an improved sanitation facility was associated with a slightly statistically significant 9% reduction in risk of diarrhea (HR = 0.91 (0.83, 0.99)). Protective effects of improved sanitation were observed and were similar in magnitude for all included species of enteric pathogens with the one exception of *Aeromonas* spp. These estimates were statistically significant at the *α* ≤ 0.05 level for two of the five virus species (astrovirus and norovirus), three of the bacterial infections (*Campylobacter*, EAEC, and typical EPEC) and for *Cryptosporidium*. Regarding the taxon-specific effects, improved sanitation afforded a moderately significant 12% reduction in risk of infection with any enteric virus (RR = 0.88 (0.82, 0.95)) and a slightly significant 9% for either protozoa (RR = 0.91 (0.83, 1.00)), and a highly statistically significant 19% reduction in the equivalent risk for any enteric bacteria (RR = 0.81 (0.72, 0.90)).

Living in a dwelling with a covered floor had the same association with the risk of diarrhea as sanitation—a moderately statistically significant 9% decrease (HR = 0.91 (0.85, 0.98)), but showed no significant associations with any enteric viral infections. Significant protective effects of improved flooring against numerous enteric bacteria species were observed ranging from a slightly statistically significant reduction in EAEC risk of 8% (RR = 0.93 (0.87, 1.00)) to a highly statistically significant 13% decreased risk of infection with *Campylobacter* spp. (RR = 0.87 (0.81, 0.94)). One of the largest effects of this or any other variable was seen for infections with *Giardia* spp., a moderately statistically significant 16% reduction (RR = 0.84 (0.76, 0.94)).

Having a primary caregiver who had completed primary education was associated with a slightly statistically significant increase in the risk of all-cause diarrhea of 7% (RR = 1.07 (1.00, 1.14)). Most other pathogen- and taxon-specific models showed a small protective effect of this exposure, which was slightly statistically significant for astrovirus (RR = 0.91 (0.85, 0.99)), any enteric virus (RR = 0.94 (0.89, 0.98)), typical EPEC (RR = 0.92 (0.86, 0.98)), *Shigella* spp./EIEC (RR = 0.92 (0.85, 0.98)), and any enteric bacteria (RR = 0.92 (0.85, 0.98)), moderately significant for *Giardia* spp. (RR = 0.87 (0.79, 0.95)), and any enteric protozoa (RR = 0.91 (0.86, 0.978)), and highly so for *Campylobacter* spp. (RR = 0.88 (0.82, 0.94)).

Living in a household with fewer than three residents per bedroom was associated with a slightly statistically significant 7% lower risk of all-cause diarrhea (RR = 0.93 (0.88, 0.99)), 5% for any enteric virus (RR = 0.95 (0.88, 0.99)), and 7% for any *Campylobacter* spp. (RR = 0.93 (0.88, 0.99)).

## 4. Discussion

During early childhood a large proportion of time is spent within the family dwelling, and therefore characteristics of the household and caregivers contribute substantially to a child’s health status, especially with regard to diarrheal disease and its sequelae. Features of the household environment may serve to promote or interrupt the transmission of diarrhea-causing pathogens via the fecal–oral and other routes. Researching these effects can be challenging, however, because studies of diarrheal disease outcomes, particularly those that make comparisons across multiple populations, may be biased by differences in caregiver reporting and understanding of diarrhea symptomatology. Detections of specific enteric pathogens from stool samples can serve as more objective, generalizable morbidity metrics for evaluating the impact of specific factors on risk of contracting disease and are increasingly being reported by trials and observational studies [39]. This was illustrated in recently published results from the SHINE trial, which showed a statistically significant decrease in parasite detections following water, sanitation, and hygiene (WASH) interventions [29] in the absence of any commensurate detectable decrease in caregiver-reported diarrhea [40]. Nonetheless, several location-specific attempts to quantify the separate effects of household characteristics on the transmission of particular enteric pathogens have yielded effect sizes that are small, non-significant, or with inconsistent directions [22,29,41].

To address these limitations this analysis combined results from several, rigorous population-based studies that each used highly sensitive, broad spectrum molecular diagnostics, and standardized exposure definitions. The resulting dataset—some 58,000 stool samples from 15,000 subjects in 22 locations—is of a size and diversity of epidemiologic contexts that is unparalleled in the study of enteric pathogens. While many of the findings appear to confirm prevailing assumptions about the transmission pathways of the analyzed pathogens, others are suggestive of hitherto novel hypotheses.

Household use of an improved water source had the largest protective effect against all-cause diarrhea of any of the five exposures. However, the direction of the association of this exposure with particular pathogens was inconsistent and, in almost all cases, non-significant. It has been suggested that sanitation interventions may be more likely to interrupt transmission of bacteria and parasites than viruses which have more environmentally mediated transmission routes [22]. However, there are known exceptions to this, and in fact, this analysis found protective effects of improved sanitation that were remarkably consistent across all pathogen species and taxa with most statistically significant effects falling within a range of around a 10–20% reduction in detection risk. While *Aeromonas* spp. infection risk was particularly reduced by improved water source, a biologically plausible finding for a pathogen that lives freely in sea and surface waters, the clinical importance of that finding is unclear. Although evidence has been published of *Aeromonas*-associated morbidity in specific contexts [42] (e.g., some South Asian GEMS sites [2]), it is not broadly believed to be an enteropathogen with a high attributable disease burden [7].

Having covered floors in the dwelling had the same effect on all-cause diarrhea as improved sanitation and to a higher level of statistical significance. Pathogen-specific estimates however show a distinct pattern of viruses being unaffected by this exposure, while consistent protective effects against bacterial and protozoal infections are evident. It is notable that the decrease in infection risk was most marked for the zoonotic enteropathogens *Campylobacter* and *Giardia*, given that many of the study sites contributing data to this analysis are in settings characterized by high levels of household-scale livestock husbandry, as well as children sharing the peridomestic space and even their sleeping quarters (the intradomiciliary space) with animals [43]. We hypothesize that investment in improved flooring materials that are easily disinfected, such as cement or tiles, can both impede the survival or transmissibility of pathogens [44] while also incentivizing the transfer of animals to predominantly outside the domestic space. For instance, the considerable decrease in infection risk for *Campylobacter* spp. can be linked to a hypothesized decrease in exposure to avian feces once improved flooring is introduced [45], as well as a decreased survival of *Campylobacter* in non-soil surfaces that are easily cleaned and dried [46,47,48]. It should be noted that some of the pathogens demonstrating risk reductions are not zoonotic in origin (e.g., *Shigella* and *E. coli* pathotypes) suggesting that improved flooring diminished transmission from human as well as animal sources.

Lack of crowding also showed a protective effect against the two zoonotic pathogens *Campylobacter* and *Giardia*. This too may be a marker of proximity to livestock, with children living in crowded households more likely to be forced to share their living or sleeping quarters with animals [43,49,50,51]. It is a limitation of this analysis that it did not include household livestock ownership among the potential exposures. This was due to heterogeneity in the ways in which this information was recorded across the different studies. The apparent increase in risk conferred by caregiver education on diarrhea of any etiology runs counter to a priori assumptions and may be an artifact of differential caregiver reporting of their child’s symptoms by education status in the MAL-ED cohorts (i.e., that mothers with higher educational attainment are more likely to report their child’s diarrheal episode to a study fieldworker when it occurs).

Another limitation inherent to the analysis of observational data, is that the role of residual confounding, in this case by socio-economic status, which can be expected to be associated with both the exposures and the outcomes, cannot be ruled out. By adjusting each of the household exposures for each other and for the two anthropometric indicators—childhood nutritional status being highly correlated with household socio-economic indicators [52]—we hoped to control for such confounding as much as possible; however, in an analysis of a dataset of this size capable of detecting small effect sizes, results should be interpreted with caution and in light of the possibility of residual confounding. Other more precise, composite indicators of household socio-economic status derived from income and asset ownership are available [52], but the necessary information was not collected uniformly across contributing studies. Future studies into this topic might consider employing causal inference methods such as propensity score weighting or matching to further adjust for confounding [53]. Another potential source of residual confounding in analyses of pooled data is differences in the ascertainment of the exposures and outcomes across the included studies due to variation in their design and the instruments used. By restricting the data to samples analyzed by highly sensitive molecular diagnostics and exposures that are widely used and reported as indicators of socio-economic development, we have attempted to minimize the possibility that such differences might lead to spurious inferences. Indeed, a sensitivity analysis (not reported) revealed single-study findings that were broadly consistent with the pooled results, taking account of the loss of statistical power from restricting the number of observations.

While systematic reviews of household-level interventions report pooled protective effects of 25–75% for diarrheal outcomes [13,54], recent single-site trials have found at best only qualified impacts [15]. The small protective effects identified in this study—reductions in risk of around 6—20% for any exposure/outcome combination—fall towards the latter, more modest end of the range of estimates. These pathogen-specific associations also appear modest in comparison to intra-subject-level exposures, such as rotavirus vaccine status—50% protective against severe rotavirus morbidity [55]—and maternal FUT2 secretor status—a 37% reduction in LT-ETEC infection [9]. Data inputs for systematic reviews skew preferentially toward efficacy-type study designs, measuring effects of delivering an intervention under trial conditions which may not be reflective of true community transmission dynamics. Apart from the 4% contributed by the SHINE trial, the data used in this analysis were from observational studies that did not seek to test an intervention. There is increasing evidence that under such real-world conditions, traditional, low-cost improvements to housing conditions (such as the installation of ventilated pit latrines) may be inadequate, and that the community is better thought of as the true unit of exposure [56]. Under this hypothesis, only an ambitious, transformative WASH agenda involving wide-ranging investments in community-level water, wastewater, housing, and livestock infrastructure and management can meet the needs for improving child health and eliminating pathogen exposure [15,56].

## 5. Conclusions

In conclusion, a diverse array of enteropathogens are endemic and highly prevalent across distinct geographical and epidemiological settings, notably *Campylobacter*, EAEC, *Giardia*, and Atypical EPEC, as has been reported elsewhere. Improvements in sanitation may reduce diarrheal disease by suppressing transmission of enteropathogens of all three taxa, while improvements in flooring may do so specifically through decreases in enteric bacteria and protozoa infection without interrupting virus transmission, with the reverse being the case for reducing household crowding. While drinking water source improvements also appear to reduce diarrhea risk, it is not clear from these findings which pathogen taxa mediate this association. By combining data collected at multiple epidemiologically and socioeconomically diverse sites, this analysis was able to quantify the associations between each of five household-level exposures on the risk of specific enteric infections, effects which differed by pathogen species and taxa but were broadly consistent with hypothesized transmission mechanisms. With further research, such estimates may be used within expanded WASH programs to target interventions to the particular pathogen profiles of individual communities and prioritize resources accordingly.

## Figures and Tables

**Table 1:** Number and percentages (%) of stool samples from children aged 0–59 months that were positive for different species of enteropathogens by qPCR in 22 study sites and overall (includes both cases and controls from the Global Enteric Multicenter Study (GEMS)1 and RECODISA).

	Adenovirus 40/41	Astrovirus	Norovirus	Rotavirus	Sapovirus	*Aeromonas* spp.	*Campylo-bacter* spp.	EAEC
Bamako, Mali	460 (26.3)	136 (7.7)	351 (19.9)	216 (12.3)	238 (13.5)	41 (2.3)	899 (50.8)	1151 (65.1)
Basse, The Gambia	373 (25.4)	98 (6.4)	250 (16.3)	227 (14.8)	201 (13.1)	19 (1.3)	787 (52.2)	873 (58.7)
Bhaktapur, Nepal	348 (5.9)	301 (5.1)	714 (12.0)	249 (4.2)	651 (11.1)	149 (2.5)	1256 (22.3)	2690 (47.0)
Cajazeiras, Brazil	1 (0.5)	9 (4.5)	8 (4.0)	12 (6.0)	9 (4.5)	6 (3.0)	6 (3.0)	68 (34.0)
Crato, Brazil	5 (2.5)	4 (2.0)	2 (1.0)	46 (23.0)	6 (3.0)	12 (6.0)	26 (13.0)	83 (41.5)
Dhaka, Bangladesh	1306 (23.5)	1142 (20.3)	1086 (19.0)	578 (10.2)	1054 (18.8)	162 (2.8)	1730 (33.8)	2245 (41.1)
Fortaleza, Brazil	137 (4.7)	52 (1.8)	187 (6.4)	40 (1.5)	132 (4.5)	40 (1.4)	401 (13.8)	883 (30.1)
Haydom, Tanzania	365 (8.3)	282 (6.4)	735 (16.6)	227 (5.2)	482 (11.0)	131 (3.0)	1818 (45.9)	2843 (65.1)
Karachi, Pakistan	366 (22.4)	191 (11.6)	393 (23.8)	161 (9.8)	306 (18.5)	122 (7.5)	1023 (62.9)	1132 (68.9)
Kolkota, India	734 (41.5)	101 (5.8)	316 (18.0)	321 (18.3)	201 (11.4)	193 (10.9)	824 (46.6)	1028 (58.1)
Loreto, Peru	1380 (21.0)	1049 (15.7)	1230 (18.0)	261 (4.0)	975 (14.9)	177 (2.6)	1293 (20.3)	3174 (55.6)
Manhiça, Mozambique	377 (36.3)	41 (4.0)	151 (14.7)	202 (19.7)	120 (11.7)	50 (4.8)	462 (44.5)	817 (78.7)
Midlands, Zimbabwe	198 (8.3)	66 (2.8)	277 (11.7)	122 (5.1)	155 (6.5)	36 (1.5)	602 (25.5)	1361 (57.6)
Mirzapur, Bangladesh	443 (24.7)	89 (5.0)	243 (13.6)	392 (21.9)	183 (10.2)	59 (3.3)	434 (24.2)	992 (55.3)
N. Feroze, Pakistan	823 (12.8)	930 (14.6)	1388 (21.4)	274 (4.2)	1063 (16.6)	35 (0.5)	1667 (27.0)	2288 (36.5)
Nyanza, Kenya	165 (9.1)	61 (3.4)	250 (13.9)	160 (9.0)	179 (10.0)	109 (6.0)	662 (36.5)	952 (53.1)
Ouricuri, Brazil	7 (3.5)	9 (4.5)	12 (6.0)	5 (2.5)	6 (3.0)	11 (5.5)	17 (8.5)	52 (26.1)
Patos, Brazil	6 (3.0)	0 (0.0)	4 (2.0)	6 (3.0)	1 (0.5)	3 (1.5)	10 (5.0)	194 (97.0)
Picos, Brazil	3 (1.8)	0 (0.0)	0 (0.0)	14 (8.5)	8 (4.9)	2 (1.0)	15 (7.5)	92 (46.0)
Souza, Brazil	2 (1.0)	0 (0.0)	10 (5.1)	3 (1.5)	5 (2.5)	2 (1.1)	12 (6.3)	161 (83.9)
Vellore, India	921 (17.1)	622 (11.6)	822 (15.2)	434 (8.1)	789 (14.7)	275 (5.1)	1144 (22.0)	3283 (62.5)
Venda, South Africa	503 (10.7)	332 (7.1)	529 (11.2)	92 (2.1)	517 (11.0)	24 (0.5)	535 (11.4)	1698 (36.0)
Total positive	8923 (15.7)	5515 (9.7)	8958 (15.6)	4042 (7.2)	7281 (12.8)	1658 (2.9)	15,623 (28.4)	28,060 (50.8)
Total stools	56,704	56,828	57,350	56,168	56,668	57,185	54,923	55,280
	**Atypical EPEC**	**Typical EPEC**	**LT-ETEC**	**ST-ETEC**	***Salmonella* spp.**	***Shigella* spp./EIEC**	***Crypto-sporidium* spp.**	***Giardia* spp.**
Bamako, Mali	382 (21.6)	588 (33.2)	511 (29.0)	319 (18.1)	52 (2.9)	567 (32.1)	522 (29.5)	1233 (70.8)
Basse, The Gambia	330 (22.0)	461 (30.8)	370 (24.8)	289 (19.3)	82 (5.5)	480 (32.7)	279 (18.5)	630 (42.4)
Bhaktapur, Nepal	1673 (28.5)	403 (6.8)	575 (9.7)	620 (10.5)	55 (0.9)	376 (6.3)	272 (4.6)	545 (10.3)
Cajazeiras, Brazil	34 (17.0)	3 (1.5)	7 (3.5)	0 (0.0)	7 (3.5)	10 (5.1)	24 (12.1)	66 (33.3)
Crato, Brazil	66 (33.0)	13 (6.5)	33 (16.5)	0 (0.0)	78 (39.0)	76 (38.0)	13 (6.5)	55 (27.5)
Dhaka, Bangladesh	1328 (23.6)	1075 (19.1)	849 (15.0)	1799 (32.6)	57 (1.0)	865 (15.4)	380 (6.8)	661 (12.8)
Fortaleza, Brazil	764 (25.9)	100 (3.4)	129 (4.4)	73 (2.5)	27 (0.9)	158 (5.4)	37 (1.3)	266 (9.7)
Haydom, Tanzania	1266 (28.6)	821 (18.6)	1141 (26.1)	1242 (28.3)	19 (0.4)	790 (17.9)	514 (12.0)	931 (27.3)
Karachi, Pakistan	358 (21.8)	545 (33.2)	404 (24.7)	356 (21.8)	22 (1.3)	543 (32.9)	383 (23.4)	931 (57.0)
Kolkota, India	519 (29.4)	367 (20.8)	392 (22.2)	293 (16.6)	23 (1.3)	533 (30.1)	275 (15.6)	1076 (62.2)
Loreto, Peru	1548 (24.1)	807 (12.1)	1137 (17.2)	763 (11.4)	85 (1.3)	786 (11.8)	633 (9.6)	1415 (26.1)
Manhiça, Mozambique	279 (26.9)	318 (30.7)	280 (27.0)	329 (31.7)	41 (4.0)	328 (31.6)	323 (31.2)	726 (70.9)
Midlands, Zimbabwe	608 (25.7)	219 (9.2)	464 (19.6)	231 (9.7)	42 (1.8)	93 (3.9)	210 (8.9)	348 (15.1)
Mirzapur, Bangladesh	430 (24.0)	209 (11.6)	337 (18.8)	95 (5.3)	22 (1.2)	648 (36.1)	80 (4.5)	377 (21.1)
N. Feroze, Pakistan	869 (13.4)	683 (10.6)	578 (8.9)	605 (9.4)	4 (0.1)	445 (6.9)	402 (6.3)	1585 (34.4)
Nyanza, Kenya	453 (25.0)	440 (24.3)	555 (31.0)	278 (15.3)	39 (2.1)	421 (23.2)	336 (18.5)	735 (40.8)
Ouricuri, Brazil	27 (13.6)	1 (0.5)	6 (3.0)	0 (0.0)	28 (14.0)	22 (11.0)	1 (0.5)	22 (11.0)
Patos, Brazil	76 (38.0)	4 (2.0)	17 (8.5)	7 (3.5)	13 (6.5)	8 (4.0)	10 (5.0)	21 (10.6)
Picos, Brazil	21 (10.5)	5 (2.5)	5 (2.5)	0 (0.0)	26 (13.0)	21 (10.5)	11 (5.5)	58 (29.0)
Souza, Brazil	86 (44.8)	3 (1.6)	12 (6.3)	5 (2.6)	17 (8.9)	10 (5.3)	17 (8.9)	30 (15.8)
Vellore, India	1440 (26.7)	870 (16.2)	877 (16.4)	717 (13.4)	67 (1.2)	701 (13.0)	278 (5.2)	1039 (23.2)
Venda, South Africa	945 (20.1)	212 (4.5)	346 (7.4)	194 (4.2)	14 (0.3)	337 (7.2)	226 (4.9)	704 (16.2)
Total positive	13,502 (23.8)	8147 (14.3)	9025 (15.9)	8215 (14.5)	820 (1.4)	8218 (14.4)	5226 (9.3)	13,454 (26.8)
Total stools	56,713	56,943	56,832	56,842	57,154	56,930	56,485	50,177

**Table 2:** Prevalence of 5 household-level exposures among study subjects aged 0–59 months in 22 study sites (before excluding samples not diagnosed with qPCR).

	Improved Water Source	Improved Sanitation	Improved Flooring	Caregiver Education	Household Crowding	Total Subjects
Bamako, Mali	5872 (87.5)	127 (1.9)	5253 (98.5)	1242 (31.4)	3119 (58.5)	6711
Basse, The Gambia	4087 (86.3)	82 (1.7)	2837 (85.0)	1029 (42.4)	2987 (89.5)	4738
Bhaktapur, Nepal	232 (98.3)	131 (55.5)	104 (44.1)	152 (64.4)	47 (19.9)	240
Cajazeiras, Brazil	172 (86.0)	197 (98.5)	198 (99.0)	137 (68.5)	-	200
Crato, Brazil	189 (94.5)	181 (90.5)	170 (87.2)	169 (84.9)	-	200
Dhaka, Bangladesh	242 (100.0)	28 (11.6)	226 (93.4)	57 (23.6)	3 (1.2)	265
Fortaleza, Brazil	142 (67.6)	201 (95.7)	208 (99.0)	146 (69.5)	6 (2.9)	233
Haydom, Tanzania	79 (31.6)	0 (0.0)	17 (6.8)	6 (2.4)	23 (9.2)	262
Karachi, Pakistan	3274 (62.6)	2428 (46.4)	2881 (75.6)	1120 (37.8)	443 (11.6)	5231
Kolkata, India	5147 (98.7)	635 (12.2)	3877 (95.9)	1880 (64.2)	160 (4.0)	5214
Loreto, Peru	309 (89.6)	70 (20.3)	98 (28.4)	192 (56.3)	45 (13.0)	378
Manhiça, Mozambique	2745 (85.1)	219 (6.8)	1691 (70.1)	454 (24.7)	417 (17.4)	3227
Midlands, Zimbabwe	611 (61.8)	619 (61.2)	547 (55.2)	844 (82.6)	-	1046
Mirzapur, Bangladesh	5907 (99.8)	2830 (47.8)	887 (20.8)	2662 (75.3)	769 (18.0)	5916
Naushahro Feroze, Pakistan	265 (100.0)	9 (3.4)	74 (27.9)	41 (15.5)	55 (20.8)	277
Nyanza, Kenya	2549 (64.5)	169 (4.3)	659 (19.5)	1630 (52.9)	34 (1.0)	3951
Ouricuri, Brazil	193 (96.5)	195 (97.5)	195 (98.0)	142 (71.0)	-	200
Patos, Brazil	199 (100.0)	197 (98.5)	198 (99.5)	139 (70.6)	-	200
Picos, Brazil	198 (99.5)	193 (96.5)	192 (96.5)	129 (64.5)	-	200
Souza, Brazil	200 (100.0)	181 (90.5)	198 (99.0)	125 (62.5)	-	200
Vellore, India	235 (100.0)	12 (5.1)	220 (93.6)	123 (52.3)	1 (0.4)	251
Venda, South Africa	216 (85.4)	3 (1.2)	233 (92.1)	207 (81.8)	62 (24.5)	314

## Supplementary Materials

The following are available online at https://www.mdpi.com/1660-4601/17/21/8078/s1, Missing data; Table S1: Percent missing data for household-level variables used in imputation by study, Table S2: Mapping of exposure variable definitions used by included studies onto published definitions, Table S3: Prevalence of time-varying covariates among study subjects at enrollment in 22 study sites.
