# Supplementary material for: Associations between Household-Level Exposures and All-Cause Diarrhea and Pathogen-Specific Enteric Infections in Children Enrolled in Five Sentinel Surveillance Studies
Source: Int J Environ Res Public Health. 2020 Nov 2;17(21):8078. doi: 10.3390/ijerph17218078 (PMC7663028; doi:10.3390/ijerph17218078)
Supplement: Supplementary file 1 [file ijerph-17-08078-s001.pdf]

Associations between household-level exposures and all-cause diarrhea and pathogen-specific enteric infections in five large surveillance studies, J. M. Colston *et al*. 2020, *International Journal of Environmental Research and Public Health*

Supplemental Materials:

Missing data:

Missing data related to the five household-level exposures were mostly due to incomplete baseline questionnaires. The main exception was the crowding variable in the SHINE study, in which information was collected on the numerator (the number of household members) but not the denominator (the number of rooms used for sleeping). Furthermore, in RECODISA the total number of rooms was recorded, but not the number used for sleeping. MAL-ED and Novel biomarkers recorded both the number of used rooms for sleeping and the number of rooms overall. Missing data for the exposures (and their components, in the case of crowding) were imputed using multivariate normal regression (MVN) with an iterative Monte Carlo method. The total number of rooms was included since it was reasoned that it would provide information as to the number of rooms for sleeping. Site, and country were also included in the imputation as predictors. Since the SHINE data lacked an entire variable component, equivalent data from the Zimbabwe Demographic and Health Surveys dating back to 2005 for the survey stratum in which the study site was located (rural Midlands province) was appended to the study database, prior to imputation to add more locally relevant information.

Table S1: Percent missing data for household-level variables used in imputation by study

	Water	Sanitation	Flooring	Education	House-hold members	Rooms for sleeping	Total rooms
**GEMS**	0.0%	0.0%	24.0%	40.7%	16.7%	24.0%	100.0%
**MAL-ED**	8.6%	8.6%	8.6%	8.6%	8.6%	8.6%	8.6%
**Novel biomarkers**	0.0%	0.0%	0.0%	5.3%	1.3%	0.0%	0.0%
**RECODISA**	0.2%	0.0%	0.7%	0.3%	0.1%	100.0%	0.1%
**SHINE**	5.5%	3.3%	5.3%	2.3%	1.5%	100.0%	100.0%

Anthropometric measurements that were not contemporaneous with the dates of sample collection were linearly interpolated and extrapolated based on the date of assessment before calculating length- and weight-for-age Z-scores. Then missing Z-scores were interpolated using the predictions from linear mixed effect models that adjusted for age with up to fourth order quadratic terms, as well as sex and allowed for site and subject specific random effects. Missing data relating to the feeding variables were imputed using predictions from Cox proportional hazard models that modeled age first of introduction of complementary foods and then of full weaning adjusting for site and sex.

Table S2: Mapping of exposure variable definitions used by included studies onto published definitions [1–3]

		Published definition	MAL-ED	GEMS	RECODISA	SHINE	Novel Biomarkers
Water source	Improved	Piped into dwelling	Piped into dwelling	Piped into house	Piped water	Piped into dwelling	Spout in the house
		Piped to yard/plot	Piped to yard/plot	Piped into yard		Piped into yard or plot	Spout on the plot
		Public tap/standpipe	Public tap/standpipe	Public tap		Piped into public tap or standpipe	Public tap/fountain
		Piped to neighbor	-	-		-	-
		Tube well or borehole	Tube well or borehole	Deep tube well<br>Shallow tube well<br>Borehole	-	Borehole	Drilled well
		Protected well	Protected well	Covered well in house or yard<br>Covered public well	Deep well	Deep well, protected<br>Shallow well, protected	Covered open well
		Protected spring	-	Protected spring	-	Protected spring	-
		Rainwater	-	Rainwater	-	Rainwater harvester	-
	Unimproved	Unprotected well	Unprotected well	Open well in house or yard<br>Open public well	Waterhole (*cacimba*)	Deep well, unprotected<br>Shallow well, unprotected	Open well without cover
		Unprotected spring	-	Unprotected spring	-	Unprotected spring	-
		Surface water	Surface water	Pond or lake<br>River or spring<br>Dam or earth pan	River, weir	Surface water (river/dam/stream/ Lake)<br>River bank/bed	Surface water (river/spring/lake)
		Tanker truck	-	-	Tanker<br>Barrel car (*carro pipa*)	Water trucking/Bowser	-
		Cart with small tank	-	-	Cart	-	-
		Bottled water	-	Bought	Mineral water	Bottled water	-
		Other	Other	Other	Other source	Other	Other

Table S2: Mapping of exposure variable definitions used by included studies onto published definitions [1–3]

		Published definition	MAL-ED	GEMS	RECODISA	SHINE	Novel Biomarkers
**Sanitation**	**Improved**	Flush - to piped sewer system	Flush to piped sewer system	Flush toilet	Public sewerage network	Flush toilet	Flush to sewer system
		Flush - to septic tank	Flush to septic tank	-	-		-
		Flush - to pit latrine	Flush to pit latrine	-	-		Flush to pit latrine
		Flush - unspecified	-	-	-		-
		Pit latrine - ventilated improved pit (VIP)	-	Ventilated VIP with water seal<br>Improved Pit latrine<br>Pour flush toilet	-	Blair Latrine (VIP)	Closed latrine with ventilated hole (VIP)
		Pit latrine - with slab	-	-	Pit (*Fossa*)	Pit latrine with slab (non-VIP)	-
		Composting toilet	-	-	-	Composting toilet	-
		Not shared	Not shared	Not shared	-	Not shared	-
	**Unimproved**	Flush - to somewhere else	Flush to somewhere else	-	-	-	Flush to somewhere else (inc. don't know)
		Flush - don't know where	-	-	-	-	
		Pit latrine - without slab/ open pit	Pit latrine without flush	Traditional pit toilet	-	Pit latrine with no slab	Hole latrine
		No facility/ bush/field	No facility/bush/ field/bucket	No facility	Without a sewerage system	No toilet or latrine of any type at the homestead	No toilet/bushes/open field
		Bucket toilet	-	-	-	-	-
		Hanging toilet/latrine	-	-	-	-	-
		Other	Other	Other	-	-	Other
		Shared	Shared	Shared	-	>1 household shares this toilet facility	Shares toilet with another household

Table S2: Mapping of exposure variable definitions used by included studies onto published definitions [1–3]

		Published definition	MAL-ED	GEMS	RECODISA	SHINE	Novel Biomarkers
Floors	Improved	Tablets/wood planks	Wood	Wood planks	Masonry	-	Wood
		Palm, bamboo	-	Palm/bamboo	-	-	-
		Mat	-	-	-	-	-
		Adobe	-	-	-	-	-
		Parquet, polished wood	-	Parquet or polished wood	-	-	-
		Vinyl, asphalt strips, floor, mat	-	Vinyl or asphalt strips	-	-	Ceramics or vinyl
		Linoleum	-	-	-	-	
		Ceramic tiles, mosaic	Ceramic tiles or vinyl	Ceramic tile	-	-	Ceramics or vinyl
		Cement	Cement/concrete	Cement	-	Concrete	Cement/concrete
		Carpet	-	Carpet	-	-	-
		Stone	-	-	-	-	-
		Bricks	-	-	-	Brick/cement	-
	Unimproved	Earth/sand/clay/mud/dung	Earth/sand/clay/mud/dung	Earth/Sand	*Taipa*[i]	Smoothed mud	Earth/sand/clay/mud
				Dung	-	Earth, sand, dung	-
		Other	Other	Other	Mixed	Sticks, wood, grass, straw	-

Table S2: Mapping of exposure variable definitions used by included studies onto published definitions [1–3]

		Published definition	MAL-ED	GEMS	RECODISA	SHINE	Novel Biomarkers
Caregiver education		Primary education: 1 – 6 years of schooling	Primary caregiver completed 6 years of education	Primary caregiver completed primary school	Mother's educational level > complete primary	Highest class completed by caregiver was standard 6 or higher	Primary caregiver completed 6 grades of education
Crowding	Numerator	Usual resident	Number of people sleeping in household	Number of people sleeping regularly in household for past 6 months	Total number of inhabitants of the house (children and adults)	Household size	Number of people sleeping in the house
	Denominator	Number of rooms used for sleeping	Number of rooms in the household used for sleeping	Number of rooms in the household used for sleeping	Total number of rooms / compartments in the house	-	Number of rooms used for sleeping

[i] In Brazil, a "*taipa*" is a small hut or dwelling constructed from adobe and other natural materials. For this analysis, it was assumed that *taipas* and mixed material dwellings had unimproved flooring whereas those made from masonry had improved flooring.

Table S3. Prevalence of time-varying covariates among study subjects at enrollment in 22 study sites

	Exclusively breastfed	Partially breastfed	Fully weaned	Moderate-severe stunting	Moderate-severe underweight	Total subjects
**Bamako, Mali**	665 (9.9)	2,885 (43.0)	3,161 (47.1)	1,223 (18.2)	1,109 (16.5)	6,711
**Basse, The Gambia**	221 (4.7)	2,294 (48.4)	2,223 (46.9)	1,212 (25.6)	1,032 (21.8)	4,738
**Bhaktapur, Nepal**	240 (100.0)	0 (0.0)	0 (0.0)	12 (5.0)	20 (8.3)	240
**Cajazeiras, Brazil**	9 (4.5)	79 (39.5)	112 (56.0)	18 (9.0)	5 (2.5)	200
**Crato, Brazil**	5 (2.5)	100 (50.0)	95 (47.5)	36 (18.0)	11 (5.5)	200
**Dhaka, Bangladesh**	265 (100.0)	0 (0.0)	0 (0.0)	44 (16.6)	48 (18.1)	265
**Fortaleza, Brazil**	233 (100.0)	0 (0.0)	0 (0.0)	6 (2.6)	9 (3.9)	233
**Haydom, Tanzania**	256 (97.7)	6 (2.3)	0 (0.0)	39 (14.9)	12 (4.6)	262
**Karachi, Pakistan**	685 (13.1)	2,069 (39.6)	2,477 (47.4)	2,528 (48.3)	2,089 (39.9)	5,231
**Kolkata, India**	749 (14.4)	2,680 (51.4)	1,785 (34.2)	1,569 (30.1)	1,093 (21.0)	5,214
**Loreto, Peru**	358 (94.7)	18 (4.8)	2 (0.5)	61 (16.1)	19 (5.0)	378
**Manhiça, Mozambique**	721 (22.3)	1,244 (38.5)	1,262 (39.1)	966 (29.9)	390 (12.1)	3,227
**Midlands, Zimbabwe**	1,046 (100.0)	0 (0.0)	0 (0.0)	94 (9.0)	65 (6.2)	1,046
**Mirzapur, Bangladesh**	1,071 (18.1)	3,424 (57.9)	1,421 (24.0)	1,523 (25.7)	1,086 (18.4)	5,916
**Naushahro Feroze, Pakistan**	164 (59.2)	110 (39.7)	3 (1.1)	74 (26.7)	61 (22.0)	277
**Nyanza, Kenya**	147 (3.7)	2,257 (57.1)	1,547 (39.2)	1,227 (31.1)	606 (15.3)	3,951
**Ouricuri, Brazil**	7 (3.5)	83 (41.5)	110 (55.0)	17 (8.5)	9 (4.5)	200
**Patos, Brazil**	6 (3.0)	73 (36.5)	121 (60.5)	23 (11.5)	8 (4.0)	200
**Picos, Brazil**	15 (7.5)	100 (50.0)	85 (42.5)	33 (16.5)	12 (6.0)	200
**Souza, Brazil**	10 (5.0)	81 (40.5)	109 (54.5)	39 (19.5)	8 (4.0)	200
**Vellore, India**	249 (99.2)	0 (0.0)	2 (0.8)	41 (16.3)	33 (13.1)	251
**Venda, South Africa**	314 (100.0)	0 (0.0)	0 (0.0)	35 (11.1)	21 (6.7)	314

References:

1. World Health Organization, UNICEF. Joint Monitoring Programme (JMP) for Water Supply and Sanitation. 2019. https://washdata.org/: Accessed: 2013 Apr 26

2. Florey L, Taylor C. Using household survey data to explore the effects of improved housing conditions on malaria infection in children in Sub-Saharan Africa. Rockville, Maryland, USA: ICF International; 2016 Aug. https://dhsprogram.com/publications/publication-AS61-Analytical-Studies.cfm: Accessed: 2019 May 30

3. Graetz N, Woyczynski L, Wilson KF, Hall JB, Abate KH, Abd-Allah F, et al. Mapping disparities in education across low- and middle-income countries. Nature. Nature Research; 2019;
